# Four antenatal care visits by four months of pregnancy and four vital tests for pregnant mothers: impact of a community-facility health systems strengthening intervention in Migori County, Kenya

**DOI:** 10.1186/s12884-024-06386-2

**Published:** 2024-03-27

**Authors:** Yussif Alhassan, Lilian Otiso, Linet Okoth, Lois Murray, Charlotte Hemingway, Joseph M. Lewis, Mandela Oguche, Vicki Doyle, Nelly Muturi, Emily Ogwang, Hellen C. Barsosio, Miriam Taegtmeyer

**Affiliations:** 1 LVCT Health, Sonning Suites, Suna Road off Ngong Rd, Adams Arcade, P.O. Box 19835, Nairobi, Kenya; 2 Department of International Public Health, Liverpool School of Tropical Medicine, Liverpool, UK; 3 Airbel Impact Lab- International Rescue Committee, Nairobi, Kenya; 4 Department of Clinical Sciences, Liverpool School of Tropical Medicine, Liverpool, UK; 5 LVCT Health, The Key Place, Along Homa Bay-Rongo Road, P.O Box 352-40300, Homabay, Kenya; 6 Kenya Medical Research Institute, P.O Box 352-40300, Kisumu, Kenya; 7 Tropical Infectious Diseases Unit, Liverpool University Hospitals Foundation Trust, Liverpool, UK

**Keywords:** Early antenatal care attendance, Health system strengthening, Quality improvement, Kenya

## Abstract

**Background:**

Early attendance at antenatal care (ANC), coupled with good-quality care, is essential for improving maternal and child health outcomes. However, achieving these outcomes in sub-Saharan Africa remains a challenge. This study examines the effects of a community-facility health system strengthening model (known as 4byFour) on early ANC attendance, testing for four conditions by four months of pregnancy, and four ANC clinic visits in Migori county, western Kenya.

**Methods:**

We conducted a mixed methods quasi-experimental study with a before-after interventional design to assess the impact of the 4byFour model on ANC attendance. Data were collected between August 2019 and December 2020 from two ANC hospitals. Using quantitative data obtained from facility ANC registers, we analysed 707 baseline and 894 endline unique ANC numbers (attendances) based on negative binomial regression. Logistic regression models were used to determine the impact of patient factors on outcomes with Akaike Information Criterion (AIC) and likelihood ratio testing used to compare models. Regular facility stock checks were undertaken at the study sites to assess the availability of ANC profile tests. Analysis of the quantitative data was conducted in R v4.1.1 software. Additionally, qualitative in-depth interviews were conducted with 37 purposively sampled participants, including pregnant mothers, community health volunteers, facility staff, and senior county health officials to explore outcomes of the intervention. The interview data were audio-recorded, transcribed, and coded; and thematic analysis was conducted in NVivo.

**Results:**

There was a significant 26% increase in overall ANC uptake in both facilities following the intervention. Early ANC attendance improved for all age groups, including adolescents, from 22% (baseline) to 33% (endline, *p* = 0.002). Logistic regression models predicting early booking were a better fit to data when patient factors were included (age, parity, and distance to clinic, *p* = 0.004 on likelihood ratio testing), suggesting that patient factors were associated with early booking.The proportion of women receiving all four tests by four months increased to 3% (27/894), with haemoglobin and malaria testing rates rising to 8% and 4%, respectively. Despite statistical significance (*p* < 0.001), the rates of testing remained low. Testing uptake in ANC was hampered by frequent shortage of profile commodities not covered by buffer stock and low ANC attendance during the first trimester. Qualitative data highlighted how community health volunteer-enhanced health education improved understanding and motivated early ANC-seeking. Community pregnancy testing facilitated early detection and referral, particularly for adolescent mothers. Challenges to optimal ANC attendance included insufficient knowledge about the ideal timing for ANC initiation, financial constraints, and long distances to facilities.

**Conclusion:**

The 4byFour model of community-facility health system strengthening has the potential to improve early uptake of ANC and testing in pregnancy. Sustained improvement in ANC attendance requires concerted efforts to improve care quality, consistent availability of ANC commodities, understand motivating factors, and addressing barriers to ANC. Research involving randomised control trials is needed to strengthen the evidence on the model’s effectiveness and inform potential scale up.

## Background

Attending at least four antenatal care (ANC) visits is essential for good maternal and child health outcomes, especially when accompanied by good quality of care [1]. Testing and early management of common antenatal conditions reduce the risks of maternal mortality and morbidity, stillbirth, low birthweight, pre-term delivery and HIV transmission [2–5]. The WHO 2016 ANC guidelines recommend starting care in the first trimester of pregnancy (12 weeks) for full ANC benefits, including HIV, anaemia, syphilis, malaria tests (in endemic zones), and supplements [6].

In Migori county, western Kenya, where this study was conducted, ANC attendance remains suboptimal despite high malaria endemicity and high HIV prevalence [7]. The county performed poorly compared to national standards in most maternal, newborn, and child health indicators. According to the 2022 Kenya Demographic and Health Survey [8], only 59% of women (aged 15–49) attended the recommended four ANC visits, even as the WHO now recommends eight ANC contacts for all pregnant women [6]; only 31% of women (aged 15–49) self-presented early enough (within the first 12 weeks of gestation) to fully benefit from testing and treatment for common pregnancy-related conditions. The delayed uptake of ANC, coupled with inconsistent availability of testing commodities limit the benefits for those who do attend, leading to delayed diagnoses of HIV, syphilis, anaemia, and malaria [9–12]. Teenage pregnancy is a concerning issue in the region, with 1 in 5 pregnant women being adolescents, who are less likely to seek timely ANC [8].

In sub-Saharan Africa, various factors contribute to delayed ANC uptake and failure to achieve the recommended number of visits, such as low knowledge of ANC benefits, stigma, financial constraints, fear of judgment/mistreatment, delayed pregnancy recognition, and limited access to quality ANC services [13, 14]. A baseline assessment conducted at the dispensary level in Siaya county, western Kenya, revealed low testing rates for malaria and anaemia (27.8%), and moderate rates for syphilis (4.3%) among ANC attendees in 2017, while HIV testing rates were almost universal (99%). However, the subsequent integration of point-of-care testing and consistent supply of testing commodities in the same sites in 2018 significantly improved completion rates for all four tests to over 95%, and ensured appropriate management for those requiring treatment [11]. This increase was achieved without disrupting existing antenatal HIV testing services or impacting waiting times or staff workload; however, late presentation remained concerning [15, 16].

Despite more women in sub-Saharan Africa now presenting for ANC at least once during pregnancy [6, 17, 18], interventions have been limited in improving early initiation, achieving four or more visits, and improving service quality. Mbuagbaw et al. [19] conducted a systematic review on the effects of health system and community interventions on ANC coverage. They identified various interventions used in low- and middle-income countries, such as financial incentives, mass media campaigns, community mobilisation, information-education‐communication, home visits by community health workers, behaviour change strategies, and policy change initiatives. However, only a few of these interventions effectively increased ANC coverage, with no single approach standing out. Since 2013, the Kenyan government has implemented free maternity policies to enhance maternal health service utilisation. Evidence indicates mixed effects of these initiatives on maternal health services, underscoring the need to combine such interventions with others addressing demand-side barriers to care and challenges in service delivery [20]. Community health volunteers (CHVs) with basic literacy and government-approved training play a crucial role in delivering maternal and child health services in Kenya by providing health promotion advice and referring pregnant mothers to ANC services during home visits [10]. Supporting CHVs in their role can lead to increased ANC uptake. For example, providing community health workers with free home pregnancy tests in a randomised controlled trial in Madagascar significantly improved pregnancy care by enabling early pregnancy confirmation and antenatal counselling [21]. Similar interventions employing quality improvement (QI) approaches at the community level in Kenya have improved skilled delivery and ANC attendance rates [22–24].

Our study aims to contribute to the discussion on effective interventions to improve the uptake and quality of ANC. This paper reports on a community health system strengthening model (called 4byFour) to increase ANC utilisation and quality. The model combines buffer stock supply and point-of-care testing for ANC, community pregnancy testing, and quality improvement strategies at the community-facility level to improve the quality and coverage of ANC. We assessed the feasibility and effects of the model on early ANC attendance, four ANC visits, and testing for four conditions by four months in Migori county, western Kenya.

## Methods

### Study design

We employed a mixed-methods quasi-experimental study with a before-after design, utilising unmatched quantitative analysis to assess the effect of the 4byFour model on the uptake of ANC and testing by four months of pregnancy, based on routine facility register data. Exploratory qualitative data was collected to enhance understanding of the findings. Our design was guided by process evaluation principles for complex interventions [25, 26], adopting a concurrent approach for triangulation through simultaneous collection of quantitative and qualitative data [27].

### Study setting and timeline

The 4ByFour model was co-developed and piloted with QI teams in two ANC facilities and their linked 6 community health units in Migori county. Migori is a predominantly rural county in western Kenya with 8 sub-counties and approximately 117 community units serving a population of about 1.1 million in 2019 [28]. The county was purposively selected on the basis of high maternal morbidity and low proportion of women attending ANC in the first trimester of pregnancy (21%) [17, 29]; and due to well-established links with the County Health Management Team and previous experience with community QI approaches in the sub-counties. Suna West sub-county was purposefully chosen by the county team for the pilot project because it had experienced previous QI programs. Site selection criteria included a high patient flow; a larger, and at least one smaller, site; as well as a site with previous QI experience. The research team conducted a situational analysis using a standard checklist in the sub-county to identify suitable sites. Arombe and God Kwer met the criteria with four and two referring community units respectively; each saw 90–120 ANC attendances per month; and both had functional community-facility QI teams. God Kwer was more rural than Arombe which was on a major road. Baseline data were collected between August-December 2019; the intervention was implemented in a phased approach with interruptions as a result of COVID-19 lockdowns between March and June 2020; endline data was collected between August and December 2020.

### Description of intervention: the 4byFour model

The 4byFour model was a community health QI approach designed to address gaps in both the demand and supply sides of the health system. The model name 4byFour describes its target of four tests (syphilis, anaemia, malaria and HIV) by four months (of pregnancy) and four (ANC) visits for all women [30]. The model was co-developed and piloted with QI teams in two ANC facilities and their linked six community health units in Migori county. Project resources were directed towards strengthening integrated point-of-care testing at the facility, community pregnancy testing and strengthening the community-facility linkage through community-facility quality work improvement teams (WITs). Traditional facility-based QI approaches were adapted to the community level to ensure they were simple, jargon-free and could be understood and implemented by integrated teams of community health volunteers and health facility staff. This adapting of QI has been suggested to be the missing piece in QI efforts in LMICs [31]. Community-facility work improvement teams brought together community health volunteers (CHVs); community members; community health assistants (CHAs), who serve as supervisors of CHVs; ANC nurse staff and the facility-in-charge of the link primary care facility. The WITS reviewed data collected at community and facility level monthly, analysed it and used it to prioritise, implement and review appropriate interventions to improve ANC attendance during the intervention. CHVs and their supervisors were trained in pregnancy mapping and the distribution and interpretation of simple urine pregnancy tests at community level [21]. During the intervention period (Feb - Oct 2020), we provided buffer stocks of rapid diagnostic test kits to the study facilities to enhance their testing capacity and avoid shortages, without disrupting the county government and KEMSA’s supply system. These facilities were equipped with HemoCue machines for haemoglobin measurement, rapid diagnostic test kits for malaria (SD Bioline Malaria Ag p.f/Pan test), and HIV/Syphilis test. Buffer stocks were provided only in the case of stock outs identified through our monthly commodity checks. Laboratory and ANC staff came up with an agreed approach to ensure testing at the point-of-care during the ANC consultation to improve availability and reduce waiting time and to record results accurately in both laboratory and ANC registers. Standard practice was to record only positive malaria results in the ANC paper register and training was given to record both positive and negative malaria tests in a spare column of the register. Supportive supervision was carried out by the sub-county health management team members quarterly to review implementation, data quality and other gaps. The research implementation team provided monthly coaching and mentorship to the WITs.

### Study populations and sample size

We included all sequential ANC attendances at the two facilities in our quantitative analysis. Using the Migori estimate prevalence of 21% of women attending ANC prior to 4 months [17] a significance level of 5% and a power of 80%, we needed to review at least 252 women’s data at baseline and endline to detect at least a 50% relative increase in the uptake of early ANC visits and testing.

Participants for the qualitative study included those directly involved as deliverers and/or beneficiaries of care i.e., pregnant mothers, community health volunteers (CHVs) and their supervisors, the Community Health Assistants (CHAs), facility staff, and senior officials of the Migori County Health Management Team. Pregnant mothers and facility staff were purposively selected from facilities where the quantitative data was abstracted, and sampled based on their experience of the intervention, willingness to participate and ability to provide consent. The CHAs, CHVs were linked to the study facilities and operated within the community health units of the facilities. The pregnant mothers were purposively sampled to represent adolescents (< 19 years) and older adults. They were approached in-person by the researchers as they visited the facility to access ANC or directly in the community. The county health officials, facility staff, and CHV/CHAs were invited (mostly by phone or in-person) to the study based on their role and interviewed if they consented. Sample size was determined by data saturation, deemed to have been reached when no new themes emerged from additional interviews [32].

### Data collection and management

#### Quantitative

Baseline data were collected from August to December 2019 and endline data collected during the same period in 2020. Data collection was impacted by interruption in intervention implementation by COVID-19 lockdown. As part of routine data collection, each ANC attendee was assigned an ANC number by the healthcare worker who completed the register. The numbers were assigned sequentially to women on their first ANC visit, considering the number of women in attendance, and the month and year of their ANC visit. ANC numbers did not follow any conventions to guarantee uniqueness. Data on ANC attendance, ANC testing, age and parity were extracted from the paper-based routine ANC registers to Microsoft Excel by a research assistant. Electronic data sets were then reviewed by facility staff from both sites until agreement was reached on the accuracy of the data. To extract data on distance to facility, we consulted the CHVs to assign a distance in kilometres to each of the village names in the visitation records. Data were double checked for accuracy. We compared clinical details (parity, age and village name) for each ANC number. For ANC numbers with different clinical details, we reviewed original paper records to make a judgement on whether the clinical details differed and ANC numbers with different clinical details were excluded from the analysis, as were records with blank or ambiguous ANC numbers.

#### Qualitative

Data were collected through individual interviews to explore the issues in greater depth and enable participants to speak openly [33]. We conducted in-depth interviews IDIs with pregnant mothers, CHVs, and facility staff at local health facilities, and key informant interviews with senior county health officials at county health offices. The interviews were carried out between November and December 2020 by experienced qualitative researchers with knowledge of the local language, culture and health system. They were conducted face-to-face and in English or Luo; lasted for about 1 h; were audio recorded and complemented with written notes. Semi-structured topic guides were used to inform the interviews; they were piloted and revised iteratively as data collection evolved. Interviews explored issues about ANC attendance, data quality, QI interventions and participants’ perception of the effects and challenges of the 4byfour intervention.

### Data analysis

#### Statistical analysis

Analysis was conducted in R v4.1.1 [34]. Descriptive statistics are medians with interquartile ranges or proportions with exact binomial confidence intervals as appropriate. Difference in patient characteristics between baseline and endline was assessed with Fisher’s exact test (categorical variables) or Kruskal-Wallace test (continuous variables). Negative binomial regression was used to test the hypothesis that the number of unique attendees increased from baseline to endline. Regression models were fitted to the number of weekly new attendees separately for the two clinics. We assessed the proportion of pregnant women who had first ANC visit before 16 weeks gestation; who had all four tests before 16 weeks gestation and who had 4 ANC visits before 36 weeks gestation. Logistic regression modelling was used to correct for the following *a priori* selected covariates: study period, clinic, age, parity and distance to clinic. We modelled the impact of patient factors on outcomes. A model including study period (baseline or endline) and clinic only as a covariate for each outcome was compared to a model including study period, clinic and all patient covariates described above (age, parity and distance to clinic) using likelihood ratio testing and the Akaike Information Criterion (AIC). A p-value < 0.05 and a lower AIC for the model including patient factors was interpreted as meaning patient factors explain some variability in outcome. Analysis of receipt of four tests was restricted to endline participants (because no participant at baseline received all four tests), and the study period variable was not included.

#### Qualitative analysis

Interviews were transcribed using a denaturalised approach and checked for accuracy and completeness [35]. The Luo interviews were translated into English. Data was analysed in Nvivo12 based on thematic framework approach. We first developed a coding framework based on a review of a sample of the transcripts, which was piloted and revised. Using the coding framework each transcript was systematically analysed to identify relevant codes, categories, and themes. An initial analysis of the quantitative data enabled the analysis to capture relevant qualitative data needed to triangulate emerging quantitative findings, including the perceived reasons for the increase in early ANC attendance, access to ANC test, and barriers to uptake of 4 ANC visits. Emerging findings were discussed among authors, feedback was obtained and subsequently integrated into the analysis.

## Results

There were 787 unique ANC numbers at baseline and 949 at endline. Among these, 80 baseline and 55 endline ANC numbers were excluded because they included participants with the same numbers but with different clinical details. This resulted in 707 baseline and 894 endline participants included in the analysis. Table 1 presents the case mix at baseline for the two clinics. Arombe had a younger age profile, but the median parity [1] and gravidae [2] were the same at both clinics, with more multiparous women attending Godkwer. Most women booked their first visit after 27 weeks gestation, and this was more common in Arombe. A minority of women (28%) attended four or more visits, and this pattern was similar at both clinics.

**Table 1:** Summary table of baseline and endline characteristics

	Baseline				Endline				Total			
	Arombe	GodKwer	Total	*p* value	Arombe	GodKwer	Total	*p* value	Baseline	Endline	Total	*p* value
N participants at baseline (3 months prior to intervention rollout and data collection)												
	369	338	707		494	400	894		707	894	1601	
**Age**												
≤ 14	7 (1.9%)	1 (0.3%)	8 (1.1%)	0.004	4 (0.8%)	7 (1.8%)	11 (1.2%)	0.153	8 (1.1%)	11 (1.2%)	19 (1.2%)	0.388
15–19	100 (27.1%)	94 (27.8%)	194 (27.4%)		137 (27.7%)	88 (22.0%)	225 (25.2%)		194 (27.4%)	225 (25.2%)	419 (26.2%)	
20–24	132 (35.8%)	101 (29.9%)	233 (33.0%)		166 (33.6%)	122 (30.5%)	288 (32.2%)		233 (33.0%)	288 (32.2%)	521 (32.5%)	
25–29	80 (21.7%)	65 (19.2%)	145 (20.5%)		98 (19.8%)	91 (22.8%)	189 (21.1%)		145 (20.5%)	189 (21.1%)	334 (20.9%)	
30–34	31 (8.4%)	39 (11.5%)	70 (9.9%)		55 (11.1%)	57 (14.3%)	112 (12.5%)		70 (9.9%)	112 (12.5%)	182 (11.4%)	
≥ 35	19 (5.1%)	38 (11.2%)	57 (8.1%)		29 (5.9%)	27 (6.8%)	56 (6.3%)		57 (8.1%)	56 (6.3%)	113 (7.1%)	
**Parity**												
Median [Min, Max]	1.00 [0, 8.00]	1.00 [0, 14.0]	1.00 [0, 14.0]	0.0538	1.00 [0, 15.0]	1.00 [0, 15.0]	1.00 [0, 15.0]	0.0298	1.00 [0, 14.0]	1.00 [0, 15.0]	1.00 [0, 15.0]	0.769
**Gravidae**												
Median [Min, Max]	2.00 [1.00, 11.0]	2.00 [1.00, 15.0]	2.00 [1.00, 15.0]	0.00425	2.00 [1.00, 16.0]	2.00 [1.00, 16.0]	2.00 [1.00, 16.0]	0.0262	2.00 [1.00, 15.0]	2.00 [1.00, 16.0]	2.00 [1.00, 16.0]	0.89
**Gestation in weeks (time at which baseline participants initiated their first ANC visit during pregnancy)**												
≤ 13	18 (4.9%)	14 (4.1%)	32 (4.5%)	0.039	41 (8.3%)	42 (10.5%)	83 (9.3%)	0.416	32 (4.5%)	83 (9.3%)	115 (7.2%)	< 0.001
14–27	138 (37.4%)	158 (46.7%)	296 (41.9%)		251 (50.8%)	188 (47.0%)	439 (49.1%)		296 (41.9%)	439 (49.1%)	735 (45.9%)	
> 27	212 (57.5%)	164 (48.5%)	376 (53.2%)		199 (40.3%)	160 (40.0%)	359 (40.2%)		376 (53.2%)	359 (40.2%)	735 (45.9%)	
**Number of baseline women making 1st, 2nd, 3rd or 4th recommended ANC visits**												
1st visit	101 (27.4%)	92 (27.2%)	193 (27.3%)	0.079	148 (30.0%)	119 (29.8%)	267 (29.9%)	0.73	193 (27.3%)	267 (29.9%)	460 (28.7%)	0.145
2nd visit	88 (23.8%)	73 (21.6%)	161 (22.8%)		116 (23.5%)	88 (22.0%)	204 (22.8%)		161 (22.8%)	204 (22.8%)	365 (22.8%)	
3rd visit	83 (22.5%)	61 (18.0%)	144 (20.4%)		110 (22.3%)	80 (20.0%)	190 (21.3%)		144 (20.4%)	190 (21.3%)	334 (20.9%)	
4th visit	57 (15.4%)	80 (23.7%)	137 (19.4%)		65 (13.2%)	63 (15.8%)	128 (14.3%)		137 (19.4%)	128 (14.3%)	265 (16.6%)	
> 4th visit	36 (9.8%)	32 (9.5%)	68 (9.6%)		52 (10.5%)	37 (9.3%)	89 (10.0%)		68 (9.6%)	89 (10.0%)	157 (9.8%)	

## Early ANC attendance

There was a statistically significant 26% increase in overall uptake of ANC across both clinics (Arombe 369 to 494 attendees IRR 1.5 [95% CI 1.1-2.0, *p* = 0.008], Godkwer 338 to 400 IRR 1.3 [95% CI 1.0-1.7, *p* = 0.048]) with more women attending for first visit before 16 weeks’ gestation: 22% (79/359) at baseline compared to 33% (119/365) at endline (*p* = 0.002) (Table 2). This increase was seen across all age groups including adolescents: 18% (21/109) of adolescents attended before 16 weeks at baseline and 32% (32/99) at endline (*p* = 0.025) (Table 2).

**Table 2:** proportion of women at baseline and endline with first or higher ANC visit at ≤ 16 weeks gestation

Age group	Baseline	Endline	*P* value
Only participants with first visit in study period			
All ages	79/359 (22%)	119/365 (33%)	0.002
Adolescents (≤ 19 years)	20/109 (18%)	32/99 (32%)	0.025
**Whole cohort**			
All ages	85/707 (12%)	152/894 (17%)	0.006
Adolescents (≤ 19 years)	20/202 (10%)	39/236 (17%)	0.049

The increase remained after correcting for changing case mix from baseline to endline in a logistic regression model as shown in Table 3 (aOR 1.69 [95% CI 1.11–2.50], *p* = 0.015). The logistic regression models including patient factors (age, parity and distance) were a better fit to the data (AIC 530.2 for patient-factor model vs. 537.8, *p* = 0.004 on likelihood ratio testing) suggesting patient factors are associated with early booking, despite the fact that the confidence intervals of the estimates of odds ratios crossed 1.

**Table 3:** Adjusted analysis and model of early ANC attendance (Association of early booking - logistic regression with linear predictors)

Variable	OR_univariable	*p*.value_univariable	OR_multivariable	*p*.value_multivariable
Endline (vs.baseline)	1.71 (1.23–2.40)	0.001	1.69 (1.11–2.58)	0.015
Age (per 5 years increase)	0.84 (0.72–0.96)	0.015	0.82 (0.62–1.08)	0.169
Parity (per 1 increase)	0.86 (0.76–0.96)	0.012	0.88 (0.72–1.07)	0.215
Distance to clinic (per 5 km increase)	1.10 (0.75–1.59)	0.608	0.98 (0.55–1.68)	0.946
GodKwer vs. Arombe	1.23 (0.89–1.70)	0.219	1.33 (0.83–2.12)	0.236

A total of 37 participants took part in the qualitative interviews. The qualitative data suggested an improved understanding of the benefits of early ANC among women after CHV visit, resulting in enhanced motivation to present early for ANC. Pregnant women reported receiving ANC education from CHVs, and many demonstrated awareness of the benefits of early ANC. Participants reported increased early detection and referral of pregnant mothers due to the community pregnancy testing, resulting in early ANC initiation: “*previously, we could only refer obvious pregnant mothers, when the pregnancy is showing, about 30 weeks gestation…. Now we can identify them early and encourage them to start early. The [pregnancy] kits have really helped* (CHV, Arombe). Several women said they were encouraged to attend ANC if a referral was backed by a positive pregnancy test: *“You feel it is urgent [to attend ANC] if the CHV tests and finds that you are positive.”* (Pregnant mother, < 18 years, Arombe). CHVs noted younger women, especially primigravida, were more receptive to the message of early ANC attendance compared with older women with previous pregnancy experience. The former appeared to be motivated by ANC testing and the need to keep their baby safe; they perceived a greater sense of insecurity and were more easily persuaded to visit ANC as a way of mitigating these risks. The latter felt they were experienced at pregnancy and childbirth. Some perceived the ANC test and iron supplements were not necessary since they had had them in their previous pregnancy.

“*The young women are eager to go; if you tell them they start clinic. But the older women feel like they can even give birth at home by themselves”* (CHV, Masara).

While women were aware of the benefits of ANC attendance some did not know the ideal gestational time for first ANC visit and the benefits of early attendance. Many still believed ANC attendance was only needed when they were ill or had experienced health challenges in their previous pregnancy: “*Coming early depends on how you are feeling and might feel that you need to go to the clinic. …you are not feeling sick or anything therefore you feel there is no need to start early”.* (Pregnant mother, 18 + years, GodKwer). Women presented late to avoid having to make many follow-on visits due to financial constraints and distance.

*“Now that we have the kits, if you confirm her pregnancy at an early stage, they fear coming to the facility because they are required to attend clinics until delivery… some stay very far away from the facility like myself who uses fifty shillings for transport, they deem that as costly if started at an earlier stage.”* (CHV, Masara).

## Availability of ANC profile tests

The project’s buffer stock improved the erratic ANC test profile supply from the national system. From February to October 2020, the project supplied more HB cuvettes, HIV/Syp DUO Kits, and Rapid Syphilis Kits than the national system (Table 4). The project supplied fewer mRDTs, causing stockouts of 41 and 53 days in Arombe and Godkwer, respectively prior to Buffer stock distributions. A 20-day stockout of HB cuvettes occurred mainly in Arombe, while Godkwer had none partly due to the project’s buffer stock. The national system did not supply any Rapid Syphilis Kits, leaving the project as the only source of 100 kits; both facilities faced 120 days of stockout for this commodity (Table 4).

**Table 4:** Status of ANC profile tests in Arombe & GodKwer health facilities from February-October 2020

Commodity	National/County supply	Project (4byFour) supply	Stockout days	
			Arombe Facility	GodKwer facility
mRDTs	825	700	41	53
HB cuvettes	50	320	20	0
HIV/Syp DUO Kits	200	450	60	45
Rapid Syphilis Kits	0	100	120	120

## Four tests by four months

At baseline no women had received all four tests by four months (16 weeks) (Table 5). Following the intervention and supply of buffer stocks this had increased to 3% (29/894). The proportion of women receiving haemoglobin and malaria testing increased to 8% and 4% respectively. These were significant increases (*p* < 0.001) but remained low due to insufficient profile tests not covered by the buffer.

**Table 5:** Proportion of women receiving diagnostic tests (Hb = haemoglobin, RPR = Syphilis. Denominator or HIV testing includes only those eligible for HIV test.)

	Proportion receiving test		
**Test**	**Baseline**	**Endline**	*p* value
**Tests before 16 weeks**			
Hb	0/707 (0% [95% CI 0–1])	70/894 (8% [95% CI 6–10])	< 0.001
HIV	42/647 (6% [95% CI 5–9])	78/836 (9% [95% CI 7–12])	0.069
Malaria	0/707 (0% [95% CI 0–1])	38/894 (4% [95% CI 3–6])	< 0.001
RPR	39/707 (6% [95% CI 4–7])	73/894 (8% [95% CI 6–10])	0.061
**All four tests**	**0/707 (0% [95% CI 0–1])**	**29/894 (3% [95% CI 2–5])**	**< 0.001**
**Tests at any time**			
Hb	0/707 (0% [95% CI 0–1])	546/894 (61% [95% CI 58–64])	< 0.001
HIV	336/647 (52% [95% CI 48–56])	489/836 (58% [95% CI 55–62])	0.188
Malaria	0/707 (0% [95% CI 0–1])	189/894 (21% [95% CI 19–24])	< 0.001
RPR	312/707 (44% [95% CI 40–48])	478/894 (53% [95% CI 50–57])	0.031
**All four tests**	**0/707 (0% [95% CI 0–1])**	**148/894 (17% [95% CI 14–19])**	**< 0.001**

**Fig. 1:**
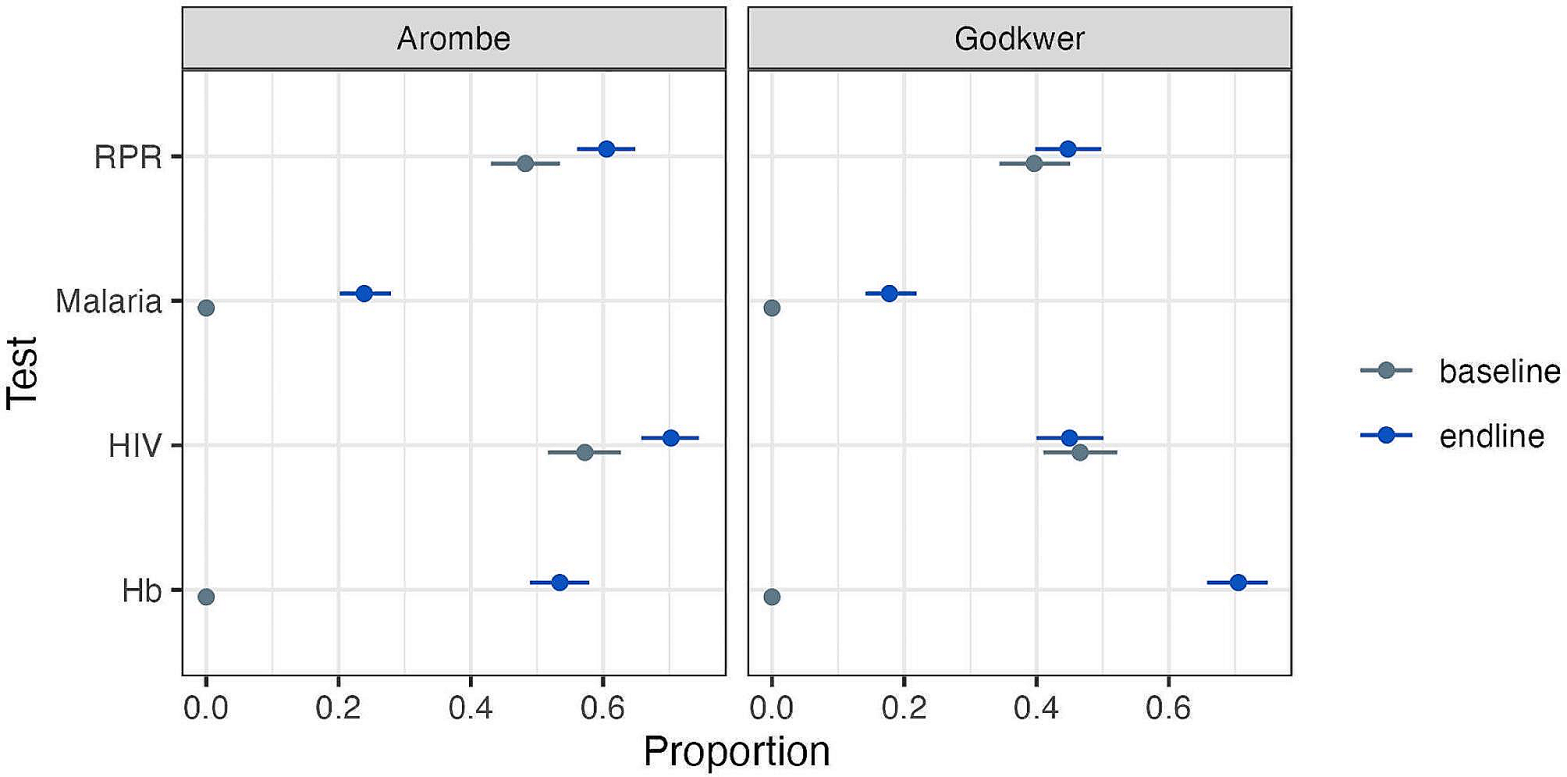
Proportion of participants receiving ANC tests at any gestation stratified by clinic

There was an overall increase in women testing driven by the increased malaria and haemoglobin testing (Fig. 1).

We carried out a post-hoc analysis of receipt of 4 tests at any gestation and showed the same pattern. No women at any gestation were recorded as having received all 4 tests at baseline and 148/894 (17%) women were recorded as receiving 4 tests at any gestation at endline. Providing enough buffer stock could have boosted test uptake significantly. Patient factors of age, parity and distance from clinic were not associated with testing (AIC for patient-factor logistic regression model 417.8 vs. 415.3, *p* = 0.315 on likelihood ratio testing, with odd ratios of effect size crossing 1 as before) (Table 6).

**Table 6:** Odds ratios of association between patient factors and testing

Name	OR_univariable	*p*.value_univariable	OR_multivariable	*p*.value_multivariable
Godkwer vs. Arombe	0.43 (0.29–0.63)	0.000	0.73 (0.41–1.29)	0.287
Age (per 5 years increase)	0.85 (0.73–0.98)	0.033	0.81 (0.59–1.09)	0.180
Parity (per 1 increase)	0.95 (0.84–1.05)	0.326	1.02 (0.83–1.22)	0.878
Distance to clinic (per 5 km increase)	0.63 (0.38–0.97)	0.049	1.04 (0.55–1.81)	0.892

Our qualitative interviews revealed the importance of a reliable supply of ANC commodities. Stockout of ANC profile commodities not covered by our buffer stock was widely reported and attributed to erratic supply by the County government. Apart from the HIV/Syphilis duo kit, the malaria RDT, syphilis rapid tests, and HemoCue cuvettes which had been out of stock for periods ranging from 2 to over 6 months when checked.

“*What has not worked well for me is the supply of ANC kits.… there is no regular supply of these kits from the County government and there is nothing you can do about it. At least when there is 4byFour program going around I will not have some of this problem challenge. I wish the County government will take charge and learn from what 4byFour is doing* (Facility staff, Arombe).

Integrated point-of-care testing was hampered by inadequate space to administer the test outside of a laboratory. Many MCH units were too small and lacked the privacy to carry out some of the tests at consultation, such as HIV and syphilis: “*Testing at the point-of-care is a good idea but the challenge for us is the space and lack of privacy”* (Facility staff, GodKwer). Further, respondents reported limited availability of MCH and laboratory staff and training on point-of-care testing, leading to delays in testing turnaround time. Other concerns related to regular power blackouts with no backup which meant laboratory tests could not be conducted.

Monthly physical checks of stock for tests and recommended treatments for each condition revealed consistent supplies for HIV testing only, with inconsistent supply from the county stores of syphilis (of HIV/syphilis duo), malaria rapid tests and the absence of cuvettes for point-of-care haemoglobin tests (using HemoCue). Drug stockouts were common. While antiretrovirals were consistently available, simple treatments including iron and folate were often unavailable.

## Four or more ANC visits

Our 4byFour model did not impact the proportion attending 4 ANC visits in pregnancy among those who would reach 36 weeks gestation during the study period (Table 7) and we found no association of 4 or more visits with patient factors (AIC 765.2 for patient factors model vs. 764.4, *p* = 0.156).

**Table 7:** Adjusted analysis and model of receipt of four or more visits

Name	OR_univariable	*p*.value_univariable	OR_multivariable	*p*.value_multivariable
Godkwer vs. Arombe	1.53 (1.19–1.97)	0.001	1.72 (1.19–2.49)	0.004
Endline (vs. baseline)	0.73 (0.57–0.94)	0.016	0.89 (0.63–1.24)	0.481
Age (per 5 years increase)	0.96 (0.87–1.06)	0.427	0.91 (0.75–1.11)	0.373
Parity (per 1 increase)	0.94 (0.87–1.01)	0.119	0.96 (0.85–1.09)	0.545
Distance to clinic (per 5 km increase)	1.29 (1.01–1.65)	0.041	1.14 (0.81–1.63)	0.445

Some providers perceived an increase in women making fourth or more ANC visits, attributed to starting ANC earlier. Other reasons included increased CHVs monitoring (and nudging) pregnant mothers and potential increased awareness of ANC benefits.

“*The uptake [of first ANC] has increased but the other… the 4th, 5th and 6th ANCs those have not been coming so much. Like when a mother has come for even 3 ANCs then they are not bothered to come for the next ones….”* (CHA, GodKwer).

*“When they start in the first month, they get many appointments, so they are able to go many times before their delivery time is due. … we visit them and remind them.”* (CHV, Arombe).

Interview data suggests women’s motivation decreased once they have finished taking the scheduled test and drugs in earlier visits. Women perceived no need to visit once the tests/supplements have been completed, especially if no health issues have been diagnosed. Additional factors included distance to the facility and lack of money for transport:

*“… they also say that “when I go there, I am going to wait for so long and after all I have gone 3 times and I didn’t have complications, I have taken IFAS and I am fine”* (Pregnant mother, 18 + years, GodKwer).

“*It is too far, and I can’t be going every month. If I go first, second and then wait closer to delivery I go again…. I have no money to travel there all the time*” (Pregnant mother, < 18 years, Masara).

## Discussion

This study assessed the feasibility and effects of the 4byFour model on early ANC attendance, four ANC visits, and four ANC tests by four months in Migori county. The model integrated existing health system models and offers a unique methodology for applying them in real life settings, advancing from ‘improvement science’ to ‘implementation science’. We found the community components of the intervention, involving pregnancy mapping, enhanced health education and referral by CHVs, significantly increased early ANC attendance among women of all ages, including adolescents. The facility-level intervention, involving buffer stock supply and point-of-care testing, increased testing overall but only marginally for women receiving four ANC tests by four months as this was determined by early attendance. The model had no effect on the proportion of women attending four or more ANC visits. The study did not yield sufficient evidence to evaluate the contribution of community-facility work improvement teams on QI and ANC uptake.

The improvement in early ANC attendance associated with community pregnancy testing and enhanced counselling and referral of pregnant mothers by CHVs is consistent with other studies showing CHW interventions increase ANC attendance [36–42]. Similar to the findings of Comfort et al. in Madagascar [43], our study demonstrates that CHVs’ distribution of pregnancy tests not only improves early detection and referral for initiating ANC at facilities but also appeared to enhance the reputation and credibility of CHVs as primary care providers. Examining these secondary effects on CHWs and their roles, as well as the socio-cultural effects on clients and communities in future research, will enrich understanding of community-based pregnancy testing. Our data also reveals the limitations of solely increasing pregnancy testing access and acknowledges other barriers to ANC utilisation that require attention. Important demand side factors such as age, parity and distance affected early ANC attendance despite the interventions, as older, multi-parous women often did not see the need to present early for ANC having gone through previous pregnancies successfully and were conscious of costs and time involved in ANC visits. Similar to findings in Uganda [44] and Rwanda [45], a multi-country study in Ghana, Kenya and Malawi found parity and age had complex impacts on ANC initiation [46]. Primigravidae were more likely to seek care early once aware of their pregnancy but less likely to recognise early pregnancy [46]. Similar to our findings, several studies have found adolescents and unmarried women delay ANC attendance due to stigma, unwanted or unplanned pregnancy or the desire to terminate the pregnancy [47]. In some communities, superstitious beliefs limit women reporting for early ANC as they do not want to disclose pregnancy status before 12 weeks for fear of pregnancy loss or curse/witchcraft [47–49]. This indicates the importance of a sensitive approach by community health workers with community pregnancy testing, counselling and referral. Implementing pregnancy testing initiatives alongside efforts to address other demand and supply-side barriers is crucial for maximum impact.

The model’s failure to improve attendance for four or more ANC visits suggests that solely increasing early ANC initiation, while proven to enhance the odds of having four ANC visits in certain cases [50], is insufficient to ensure consistent or four ANC visit attendance. Accessibility challenges, such as distances to facilities and financial constraints, were widely reported to affect subsequent ANC visits after initiation and aligns with findings across LMIC contexts [44, 45]. Behavioural factors, such as women’s limited understanding of the preventive value of ANC and the benefits of follow-on attendance (beyond the first ANC visit), were equally pertinent. Cultural, spiritual beliefs, personal issues, and variable ANC service quality in health facilities can impact ANC attendance [51, 52]. Quality of care factors, including infrastructure, commodities, supplies, and health worker skills and attitudes, affect ANC visits in most LMIC contexts [53]. Inequalities in care quality have been noted in certain settings, indicating their potential impact on disparities in ANC attendance. A study in Kenya found the youngest, poorest, least educated, most disadvantaged, and most disempowered women are most likely to report poor experiences of care [54]. This suggests sustained patient-centred QI efforts are needed to address health inequalities and improve ANC attendance. While Kenya’s Linda Mama initiative offers free maternal and child health services, coverage is incomplete, and it does not cover transportation costs [55]. Decentralising ANC by training community health workers to provide low-risk antenatal care at the community level, such as distribution of IFAS and IPTp, and pregnancy testing, could reduce the distance barriers [41].

The low uptake of four test by four months partly results from poor ANC attendance in the first trimester, when most tests were done as per national guidelines. Additionally, we observed major procurement and supply chain issues for anaemia testing, malaria rapid tests and iron/folate supplements, which may have hindered the model’s impact on early and 4 ANC visits. County stockouts prevented four tests from being done, which discouraged women from attending subsequent ANC visits. Even when test commodities were available, other factors such as human resource shortages, lab testing, and inconsistent recording of malaria results limited the effect of the increased commodity availability for test uptake. Lab tests were affected by power blackouts, while point-of-care tests were affected by lack of privacy and confidentiality. HIV testing and antiretroviral therapy were consistently high and unaffected by the intervention, indicating their support from vertical programmes compared to other ANC elements. Stockouts of essential commodities are a significant challenge in ANC and highlight the fragmentation of supply systems along vertical disease programmes [56]. Several studies have reported that commodity stockouts discourage pregnant women from attending ANC in Africa [57–59], although there is limited evidence on the effects of buffer stock interventions on ANC attendance. Nonetheless, our provision of buffer stock for essential commodities improved ANC test uptake and quality care by smoothing out stock issues, demonstrating the critical importance of sustained commodity availability in ANC utilisation beyond donor funded projects and research. Buffer stock alone could have produced similar intervention outcomes. Thus, effective ANC requires integrated supply chains to ensure availability of core primary care essential commodities [60, 61]. Core treatment for common conditions such as anaemia or malaria may be overlooked by top-down programmes from large multilateral organisations, as seen in studies in Tanzania, Zambia and elsewhere where the well-funded HIV program reduced ANC clinic attendance and testing of other conditions [50, 62, 63].

Similar to prior findings [64], our study identified significant data quality issues, including incomplete and inaccurate ANC registers, a lack of unique patient identification for tracking, data fragmentation among registers, and disconnected health data between community and facility levels. Digitised approaches to data collection at both community and facility levels could potentially address these challenges, but long-term sustainability beyond project funding is imperative [65]. The Kenyan MoH has recognised the potential of digitised health data to tackle data quality concerns, culminating in the launch of a costed strategy to guide a fully national electronic Community Health Information System (eCHIS), piloted in Kisumu County [66] and now being rolled out across the country. Establishing community-based ANC to complement facility-based digital ANC records and creating sustainable linkage between these platforms are essential steps to help Kenya achieve WHO’s ambitious goal of eight ANC contacts.

The QI approach of the 4byFour pilot was shown to work to improve CHV pregnancy testing, referral and linkage to health facilities (demand side). The intervention was based on a health system strengthening approach and focused on improving existing systems and resources to optimise ANC service delivery, rather than introduce new elements. During the 4byFour pilot, the local implementing partner, research team, county health team, community health volunteers and facilities worked together to co-design the intervention aiming to work within and maximise the existing capacity of the system to promote sustained quality improvement. However, it faced numerous sustainability challenges of testing procurement and supply chain, workforce capacity, and intersecting vertical programs demonstrating the need to effectively address both supply and demand side factors to effectively achieve ANC outcomes. Sustainability of QI interventions beyond project funding is essential to strengthen health systems and deliver lasting improvement in maternal and child health outcomes [67].

## Strengths, limitations, and future research

This study offers valuable insights into the potential effects of combining various health system strengthening approaches on antenatal care attendance, while providing useful insights on the individual components of the model. However, it has some limitations. The before-after design limits our ability to rule out other factors that may have caused the observed changes from baseline to endline. Data quality issues from paper-based ANC registers extraction may compromise data reliability, despite data review by facility staff. Budget constraints hindered the buffer stock intervention from addressing all essential commodity stockouts, possibly affecting the model’s effectiveness. The cross-sectional design limits causal inferences from participants’ experiences. Future research using longitudinal and randomized controlled trials will enhance the evidence on the model’s impact. Moreover, a cost-effectiveness analysis and an examination of contextual factors influencing the model’s outcomes will be useful in informing future scale-up efforts. There is the need for innovative approaches to assess the potential effect of the QI component of the model on ANC uptake.

## Conclusion

This study demonstrates the potential of the 4byFour model to improve ANC coverage in resource-poor health systems. The model increased ANC uptake, especially early ANC attendance among all age groups, including adolescents who usually engage less in care during pregnancy. The model also improved essential ANC testing for malaria, HIV, syphilis, and haemoglobin. Community pregnancy testing and buffer stock provision of ANC profile tests had particularly promising results. The findings suggest that the 4byFour model and its components, such as community pregnancy testing and buffer stock provision of ANC commodities, can be used to tackle low and delayed ANC uptake and quality issues. Sustained improvement in ANC attendance requires a concerted effort to improve quality of care and availability of ANC commodities, understand motivating factors and barriers to ANC, and promote incentives for horizontal investment in health system strengthening that prioritises integrated patient-centred care over fragmented verticalisation. Further research using longitudinal and randomised control trials is needed to strengthen the evidence on the model’s effectiveness and scale up.

## Data Availability

Data are available from the corresponding author on request.

## Abbreviations

ANC: Antenatal Care
CHAs: Community Health Assistants
CHVs: Community Health Volunteers (now known as Community Health Promoters)
HIV: Human Immunodeficiency Virus
LMICs: Low-and Middle-Income Countries
QI: Quality improvement
WHO: World Health Organization
WITs: Work Improvement Teams
